# Detection of Cardiac Function Abnormality from MRI Images Using Normalized Wall Thickness Temporal Patterns

**DOI:** 10.1155/2016/4301087

**Published:** 2016-03-01

**Authors:** Mai Wael, El-Sayed H. Ibrahim, Ahmed S. Fahmy

**Affiliations:** ^1^Nile University, Juhayna Square, Shiek Zayed, Cairo 12588, Egypt; ^2^University of Michigan, 1500 E. Medical Center Drive, Ann Arbor, MI 48109, USA; ^3^Biomedical Engineering Department, Cairo University, Cairo, Egypt

## Abstract

*Purpose*. To develop a method for identifying abnormal myocardial function based on studying the normalized wall motion pattern during the cardiac cycle.* Methods*. The temporal pattern of the normalized myocardial wall thickness is used as a feature vector to assess the cardiac wall motion abnormality. Principal component analysis is used to reduce the feature dimensionality and the maximum likelihood method is used to differentiate between normal and abnormal features. The proposed method was applied on a dataset of 27 cases from normal subjects and patients.* Results*. The developed method achieved 81.5%, 85%, and 88.5% accuracy for identifying abnormal contractility in the basal, midventricular, and apical slices, respectively.* Conclusions*. A novel feature vector, namely, the normalized wall thickness, has been introduced for detecting myocardial regional wall motion abnormality. The proposed method provides assessment of the regional myocardial contractility for each cardiac segment and slice; therefore, it could be a valuable tool for automatic and fast determination of regional wall motion abnormality from conventional cine MRI images.

## 1. Introduction

**Figure 1:**
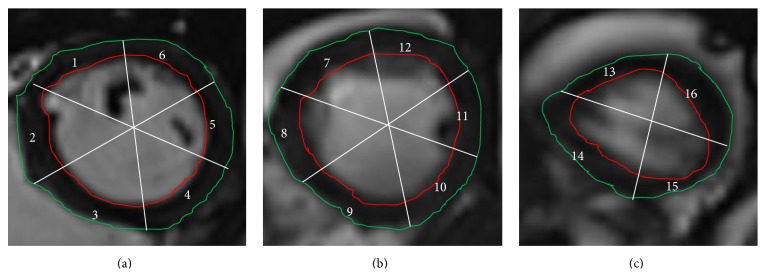
Segmentation example in a normal case showing (a) basal, (b) midventricular, and (c) apical slices.

Imaging the heart using cine magnetic resonance imaging (MRI) is a powerful tool for assessing cardiac global and regional functions [1]. In cine MR imaging, a series of cardiac images are acquired at different cardiac phases (about 25 timeframes per cardiac cycle) [2]. The imaging sequence is repeated several times to image multiple cross sections of the heart, for example, basal, midcavity, and apical short-axis planes, as shown in Figure 1. The first step to assess the cardiac function is to delineate the inner and outer myocardial contours, namely, the endo- and epicardium, of the left ventricle (LV). To assess global heart function, parameters such as the LV volume, mass, and ejection fraction (EF) are calculated using the areas/volumes enclosed by the different contours [1]. In many cases, global functional parameters cannot reflect subtle wall motion abnormalities. In such cases, assessment of regional cardiac wall motion is required. The latter is usually done by visual evaluation of the heart wall motion [3], which is highly subjective and requires experienced graders. Therefore, a quantitative method for assessing regional wall motion abnormalities is needed. While tagged MRI might provide such quantitative measurements, the technique is not usually implemented on a clinical routine basis because, in addition to the extra scan time, it requires sophisticated and time-consuming analysis [4].

Several methods have been proposed for the quantification of the myocardial wall motion from standard cine MRI images. These methods can be classified into intensity-based and contour-based methods. Intensity-based methods utilize the fact that the blood pool signal intensity is much higher than that of the myocardium, and thus the myocardium contraction is accompanied by a significant change in the overall intensity within a neighborhood around the heart. For example, the intensity histogram at each cardiac phase has been used as a key feature to represent the intensity variations within a fixed area enclosed by the epicardium contour of the initial timeframe [5]. Another example includes segmentation of the blood cavity at each cardiac phase using simple thresholding method, then using its area as a feature to detect motion abnormality [6]. Nevertheless, the intensity-based methods are limited by a number of factors. First, the assumption of high blood signal intensity makes such techniques inapplicable for black-blood images, for example, those acquired with stimulated echo acquisition mode (STEAM) sequences [7]. Further, the intensity-based features are not reliable for processing low-quality images. Finally, the resulting quantities calculated from the blood cavity do not represent the myocardial thickness and thickening. Such parameters are of utmost clinical importance as they are affected by altered regional myocardial function.

The contour-based methods can avoid some of the abovementioned limitations. They start off by (manually) identifying the myocardium contours at two or more cardiac phases to detect wall motion abnormalities. In one method, these contours are used to build a finite element model representing the LV deformation at two cardiac phases: end diastole (ED) and end systole (ES) [8]. Based on this model, global variation of the LV function is measured by estimating longitudinal shortening, wall thickening, and twisting. Nevertheless, this approach does not provide information about the specific location of wall motion abnormality. One solution, though, is to use statistical shape analysis to detect wall motion abnormality [9]. Nevertheless, this approach has another drawback that the implemented statistical model is usually built from normal cases only, which makes it not suitable for accounting for potential overlapping between different pathologies and normal wall motion patterns. In such cases, analysis of the wall motion pattern during the whole cardiac cycle may be necessary [10], although information about the location of the regions with abnormal wall motion, as well as information about wall thickening, is still missing [8, 10].

The aim of the proposed work is to develop a technique that can capture the variations in myocardial thickness during the cardiac cycle and provide an accurate method for assessment of regional myocardial wall motion from nontagged cine MRI images. Using a dataset of normal and abnormal cases, segmented manually, we extract regional changes in myocardial thickness during the whole cardiac cycle based on standard American Heart Association (AHA) 17-segment model [11]. The extracted thickness pattern is normalized relative to the average radius of the epicardium. The resulting patterns are then mapped to lower dimensions using principal component analysis (PCA). The last step of the processing algorithm is feature classification using the maximum likelihood (ML) criterion with leave-one-patient-out method [12]. It is worth noting that although the normalized LV thickness is used as a clinical measure in echocardiography [13, 14], it has not been used before for the assessment of cardiac MR images.

## 2. Methods

### 2.1. Dataset and Image Preprocessing

An image dataset from 14 normal subjects and 13 patients (4 with myocardium infarction (MI), 5 with pulmonary hypertension (PH), and 4 with hypertrophic cardiomyopathy (HCM)) was used to train and test the proposed method. Three short-axis (SAX) slices (basal, midventricular, and apical), each with 23–25 timeframes, were acquired for each subject using standard cine steady-state free precession (SSFP) pulse sequence [13], resulting in total of 1863 image. The cine images were acquired using the following imaging parameters: matrix = 320 × 320, resolution = 1.13 × 1.13 mm^2^, slice thickness = 8 mm, flip angle = 60°, and repetition time (TR)/echo time (TE) = 2.8/1.4 ms, readout bandwidth = 1140 Hz/pixel, parallel acceleration factor = 2.5, views per segment (turbo factor) = 12, and # averages = 1. Only the first 23 timeframes in all cases were processed for consistency. In addition to the cine images, delayed enhancement (DE) MRI [15] images were acquired for the patients with MI. This data was used to validate the ability of the proposed method to identify regional wall motion abnormality. The imaging parameters of the DE images were matrix = 576 × 576, resolution = 0.625 × 0.625 mm^2^, slice thickness = 8 mm, flip angle = 25°, and TR/TE = 5.4/2.48 ms, readout bandwidth = 1140 Hz/pixel, parallel acceleration factor = 2.5, views per segment (turbo factor) = 19, and # averages = 3.

All the images were manually segmented to extract the epicardium and endocardium. The papillary muscles were excluded from the myocardium. Each contour was then resampled at equiangular spaces to a standard vector length of 60 points in the mid and basal slices and 40 points in the apical slices. A fixed anatomical landmark was defined at the intersection between the right ventricle and septum to identify the location of each segment.

### 2.2. Normalized Wall Thickness (NWT)

At any given timeframe, wall thickness was calculated at the *k*th contour point as the radial distance connecting the *k*th point on the endocardium to the corresponding one in the epicardium, as previously explained [9]. The myocardial wall thickness was then normalized by dividing the calculated distances by the mean radius of the epicardium (at the initial timeframe). This step is needed to avoid interpatient variability due to different heart sizes.

#### 2.2.1. Segmental NWT

**Figure 2:**
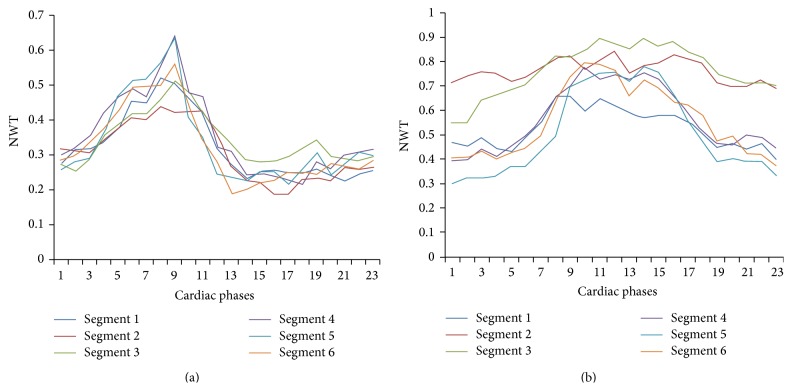
Normalized wall thickness (NWT) throughout the cardiac cycle for all segments in a midventricular slice from (a) normal volunteer and (b) patient with hypertrophic cardiomyopathy (HCM).

The three SAX images in each dataset were segmented by a faculty in radiology with 13 years of experience in cardiac MRI using the QMass software (Medis medical imaging systems, Leiden, Netherlands) based on the AHA 17-segment model [11]. The apical cap was removed as no useful segmentation results are available from this segment. The normalized wall thickness values of all contour points within each segment were averaged to represent each segment by one normalized wall thickness (NWT) value. That is, each segment is represented by a NWT vector, *t*_*i*,*j*_ representing the average thickness of the *i*th segment (*i* = 1 : 16) at different 23 cardiac phases (or timeframes). The subscript *j* represents the *j*th patient (*j* = 1 : 27).  Figure 2 shows an example of normal and abnormal NWT patterns in a midventricular SAX slice.

### 2.3. Feature Vector and PCA

#### 2.3.1. Training

The leave-one-patient-out method [12] was applied to build 16 independent elements training data for each segment position of *N* − 1 case, where *N* is the number of cases in the dataset (*N* = 27). That is, one of the 27 cases is selected to perform the test while the remaining datasets are used for training. The process is then repeated by selecting another test case and performing the training using the remaining cases, and so forth until all the 27 cases are tested. The error mean and standard deviation resulting from the 27 cases is then calculated. For each of the 16 segments, all the NWT vectors of the 26 training subjects *t*_*i*_were used to construct a matrix *T* (of size = 23 × 26), as follows:

(1) $$\begin{array}{c} T=\left[\;t_{1}\;|\;t_{2}\;|\;\cdots \;|\;t_{N-1}\;\right]. \end{array}$$

PCA was applied on the *T* matrix to find the directions of the data variations, which correspond to the eigenvectors of the covariance matrix: COV = *T* · *T* ^*T*^ [16]. The first *n* eigenvectors, *v*_*j*_, corresponding to the maximum *n* eigenvalues, are then stacked to form the matrix *V* representing the directions of the data variation and their associated eigenvalues *e*

(2) V=v1 ∣ v2 ∣ ⋯ ∣ vnT,e=e1,…,enT.

The feature vectors that represent the contraction pattern of a given segment are then created by projecting its NWT vector, *t*_1_, on the subspace formed by the obtained *n* eigenvectors; that is,

(3) $$\begin{array}{c} F=V\cdot T, \end{array}$$

where *F* is an *n* × *N* − 1 matrix whose *i*th column represent the feature vector of the *i*th subject. The number of principal components, *n*, was selected experimentally as will be discussed below. Repeating the training phase for all the myocardium segments yields 16 sets of features vectors, each will be used to build a classifier as will be discussed below.

#### 2.3.2. Testing

Given a NWT vector, *t*, of the *j*th segment, the feature vector, *f*_*j*_, is created by projecting the vector *t* on the selected *n* eigenvectors; that is,

(4) $$\begin{array}{c} f_{j}=V\cdot t. \end{array}$$

Then, the vector *f*_*j*_ (length = *n* × 1) is fed to Naïve Bayes' classifier to determine whether the *j*th segment is normal or abnormal as described below.

### 2.4. Classification

Naïve Bayes' classifier was applied to assign a label, *c*, to each segment (normal or abnormal) [17], where equal prior probability was assumed for both classes. That is, given a vector, *f*_*j*_, then segment *j* is assigned the label *c* that maximizes the likelihood function *P*(*f*_*j*_∣*C* = *c*). Assuming a multivariate Gaussian distribution [13], the latter is given by

(5) Pfj ∣ C=c;μ,Σ=12πn/2Σ2exp⁡−12fj−μTΣ−1fj−μ,

where *μ* and Σ are the mean vector and covariance matrix of the feature vectors of the training set, that is, columns of the matrix *F*, representing class *c*.

### 2.5. Experiments

#### 2.5.1. Experiment 1

This experiment was designed to determine the minimum value of parameter “*n*” (the number of principal components) that achieves the best performance. First, the classification process was conducted using *n* = 1 and the performance was measured using the *F*1 score, defined as follows [18]:

(6) $$\begin{array}{c} F1=\frac{2TP}{\left(2TP+FP+FN\right)}, \end{array}$$

where the true positive (TP), false positive (FP), true negative (TN), and false negative (FN) values are calculated using the leave-one-patient-out method for each segment with clinical diagnosis as the gold standard. The experiment was then repeated for *n* = 2,3. The *F*1 score for each value of *n* was recorded, and the value that yielded the maximum score was used for final classification.

#### 2.5.2. Experiment 2

After determining the optimal *n* value from Experiment 1, a second experiment was conducted to evaluate the overall performance of the proposed method using the leave-one-patient-out method. Classification was applied using 16 independent classifiers representing different segment location; slice abnormality was determined based on the results gathered from the 16 classifiers by determining the number of abnormal segments at each slice, where a slice was considered abnormal if it contained one or more abnormal segments.

The algorithm's sensitivity, specificity, and accuracy for identifying patients (with abnormal contraction pattern) were determined using the following equations, based on the clinical diagnosis of these patients:

(7) Sensitivity=TPTP+FN,Specificity=TNTN+FP,Accuracy=TP+TNTN+FN+TP+FP.

In addition, for patients with DE images, the hyperenhanced segments from the DE images were compared to those identified by our method as abnormal.

## 3. Result

The results of* Experiment 1* are summarized in Table 1, where the *F*-score of the classification process is listed for one, two, and three principal components. As can be shown, the highest *F*-score (i.e., highest accuracy) occurred when only one principal component was used. Consequently, the parameter *n* was set to 1 in Experiment 2. It is worth noting that in this experiment the largest component captured about 89% of all data variations.  Table 2 summarizes the results obtained from* Experiment 2*, where 92% true positive (TP) and 79% true negative (TN) were achieved for the basal slices, and 77% TP for the mid and apical slices. The sensitivity and specificity for each slice level are also illustrated in the table. The calculated overall system accuracy was found to be 81.5%, 85%, and 88.5% for the basal, mid, and apical slices, respectively.

**Figure 3:**
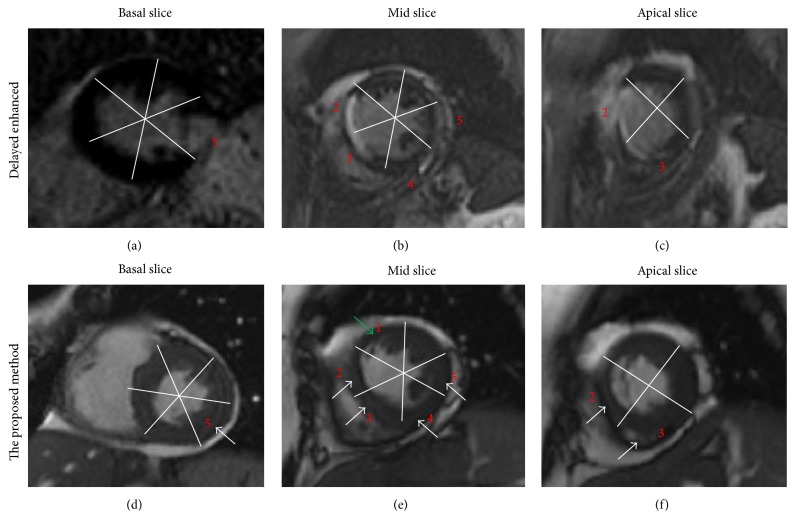
Comparison between (a–c) infarcted regions using delayed enhancement (DE) MRI and (d–f) regions with motion abnormality detected using the proposed method.

The hyperenhanced segments in the DE images (first row in Figure 3) correspond to the abnormal segments (pointed to by white arrows in the figure) detected by the proposed method. However, it can be shown in the figure that some segments (e.g., segment 1 in Figure 3(e), indicated by green arrow) are classified as abnormal while it has normal intensity in the DE image (Figure 3(b)). This may be explained by the fact that the DE images show only the infarcted regions, not regions with abnormal wall motion (e.g., the peripheral zone).

## 4. Discussion

We proposed a novel feature vector, namely, the normalized wall thickness, which can be used to detect wall motion abnormality. This feature considers the variations between normal and abnormal contraction by tracking the normalized thickness of all segments between the endo- and epicardium during the whole cardiac cycle. The proposed method provides a simple tool for the assessment of the regional abnormality for each segment in each slice; therefore, it could be a valuable tool for automatic and fast determination of regional wall motion abnormality from conventional untagged cine images.

The proposed method's higher specificity relative to sensitivity reflects its tendency for true identification of normal cases over abnormal cases. Among the different groups of patients, it was observed that the proposed method was able to detect the abnormal cases with MI and HCM with better accuracy than the cases with the PH. This could be explained by the fact that PH is manifested mainly by RV, rather than LV, remolding. This capability could be added to the current algorithm using the Lunar Index, as previously described [19].

One limitation of the proposed study is the small number of studied subjects. However, we focused in this pilot study on proofing the concept of work of the new proposed technique. Future steps include collaboration studying a larger number of subjects with different disease stages and stratifying the results based on heart disease to confirm the results in the current study.

## Figures and Tables

**Table 1:** The effects of the number of principal components (PC) on *F*-score for 3 slice levels: basal, mid, and apical.

Slice	1-PC	2-PC	3-PC
Basal	0.84	0.72	0.75
Mid	0.80	0.8	0.8
Apical	0.87	0.73	0.73

**Table 2:** Classification results according to one principal component, values represented as percentages.

Slice	TP	FP	TN	FN	Sensitivity	Specificity	Accuracy
Basal	92	8	79	23	92%	77%	85%
Mid	77	23	86	14	77%	86%	81.5%
Apical	77	23	100	0	77%	100%	88.5%

FN, false negative; FP, false positive; TN, true negative; TP, true positive.
